# Anticancer effects of miR-124 delivered by BM-MSC derived exosomes on cell proliferation, epithelial mesenchymal transition, and chemotherapy sensitivity of pancreatic cancer cells

**DOI:** 10.18632/aging.103997

**Published:** 2020-10-11

**Authors:** Yan Xu, Nanbin Liu, Yuhua Wei, Deren Zhou, Rui Lin, Xiuyan Wang, Baomin Shi

**Affiliations:** 1 Department of General Surgery, Tongji Hospital, Tongji University Medical School, Shanghai 200065, China; 2 Department of Ultrasonography, Tongji Hospital, Tongji University Medical School, Shanghai 200065, China

**Keywords:** cancer, miR-124, BM-MSCs, chemotherapy sensitivity, epithelial mesenchymal transition

## Abstract

Objective: This study aims to explore the roles of miR-124 in pancreatic tumor and potential vehicles.

Results: The miR-124 expression levels decreased in pancreatic adenocarcinoma tissues and cancer cell lines AsPC-1, PANC1, BxPC-3 and SW1990. Furthermore, the elevated expression of miR-124 in AsPC-1 and PANC1 *via* miR-124 mimic transfection-induced apoptosis, metastasis and epithelial mesenchymal transition was suppressed, and the EZH2 overexpression partly reversed the protective effects of miR-124 against pancreatic tumors. In addition, the expression of miR-124 was detected in exosomes extracted from miR-124-transfected BM-MSCs, and these exosomes delivered miR-124 into pancreatic cancer cells, and presented the anti-tumor effects *in vitro* and *in vivo*.

Conclusion: MiR-124-carried BM-MSC-derived exosomes have potential applications for the treatment of pancreatic tumors.

Methods: The expression of miR-124 and EZH2 was determined in both pancreatic cancer tissues and cell lines. miR-124 or EZH2 was overexpressed in AsPC-1 and PANC1 cells. Then, the effects on cell viability. apoptosis, invasion, migration and epithelial mesenchymal transition were evaluated. Afterwards, the roles of miR-124 on the expression and function of EZH2 in pancreatic tumors were determined by dual luciferase reporter assay. Subsequently, miR-124 was transfected to bone marrow mesenchymal stromal cells (BM-MSCs), and the BM-MSCs derived exosomes were isolated and co-cultured with AsPC-1 and PANC1 cells, or injected into pancreatic cancer tumor-bearing mice.

## INTRODUCTION

Pancreatic cancer, particularly pancreatic ductal adenocarcinoma (PDAC), is one of the main threats for public health, although pancreatic cancer is not the most common cancer type. The latest global cancer statistics have illustrated that the incidence of cancer at the pancreas only accounted for 2.5% of total new cancer cases, and this incidence has remained stable for the past three decades [1, 2]. The major problem is that pancreatic tumors have high fatality, and have a 5-year survival ratio of approximately 5-6% [1]. Furthermore, the mortality of pancreatic cancer accounted for 4.5% of all cancers, which is almost double its incidence [2]. One fifth of pancreatic cancers cases occur in China, among which nearly 40% were under 65 years old [3]. In addition, 90% of pancreatic cancers have mutations in K-Ras. Unfortunately, to date, there is still no K-Ras-target drug for therapy. The best strategy for pancreatic cancer therapy is surgical resection, as long as it can be detected early, because surgery provides good short-term outcomes. However, there is a real challenge for pancreatic cancer diagnosis, because this presents with very few typical symptoms [1, 4]. Recent progresses of laparoscopy have provided a strong tool for pancreatectomy. However, merely few pancreatic cancer patients are suitable for surgery. The reason for this is that many patients presented with transforming diseases when upon diagnosis, which lead to poor prognosis [1, 5]. In addition to surgery, chemotherapy, such as Gemcitabine and Fluorouracil (5-FU), have been used for pancreatic cancer treatment. However, chemoresistance limits the outcomes [6]. Hence, there is need to identify new weapons against pancreatic cancer.

Non-coding RNAs, particularly microRNAs (miRNAs), have been verified by numerous recent researches to be critical molecules involved in the progression of pancreatic cancer [7–9]. MiRNAs are short single-strand RNAs within typical 20-24 nucleotides processed from pri-miRNA and pre-miRNA, and has the ability to modify the target gene expression. In mammals, such as humans, mature miRNAs inaccurately and complementarily bind to the 3’ untranslated region (UTR) of the target mRNAs, and modestly repress the gene expression [10]. In cancer, miRNAs induce a crucial effect on all kinds of procedures, such as cancer cell proliferation, connection, metastasis and apoptosis. This has been considered as a potential target for the diagnosis and treatment of tumors [11]. Among the thousands of miRNAs, miR-124 has attracted increasing attention in pancreatic cancer. Some clinical studies have found that miR-124 expression decreases in pancreatic tumor patients, and K-Ras has been identified as an immediate goal of miR-124 [12, 13]. Furthermore, EZH2 is a histone methyltransferase that catalyzes the methylation of H3K27me3 in pancreatic tumors, and has been considered as a downstream gene of K-Ras. In addition, EZH2 has been considered to be critical for pancreatic cancer [14, 15]. Interestingly, EZH2 has been considered as a target of miR-124 in gastric tumors [16]. However, it remains unclear whether the expression of EZH2 is regulated by miR-124 in pancreatic cancer, and what the underlying mechanisms are.

Exosomes are the vehicles of extracellular miRNAs, and exosomal miRNAs take part in the metastasis and chemoresistance of cancer cells [17]. Tumor-derived exosomes may even repress the immune response against cancer [18]. Exosomes are 30-100 nm extracellular vesicles that contain mRNAs and miRNAs. These are found in body fluids, such as blood, urine and saliva [19]. The present study aimed to identify the new target gene of miR-124, and explore the function of exosomal miR-124 in pancreatic cancer treatment.

## RESULTS

### MiR-124 expression was downregulated and the expression of EZH2 was stimulated in pancreatic cancer tissues and cell lines

**Figure 1:**
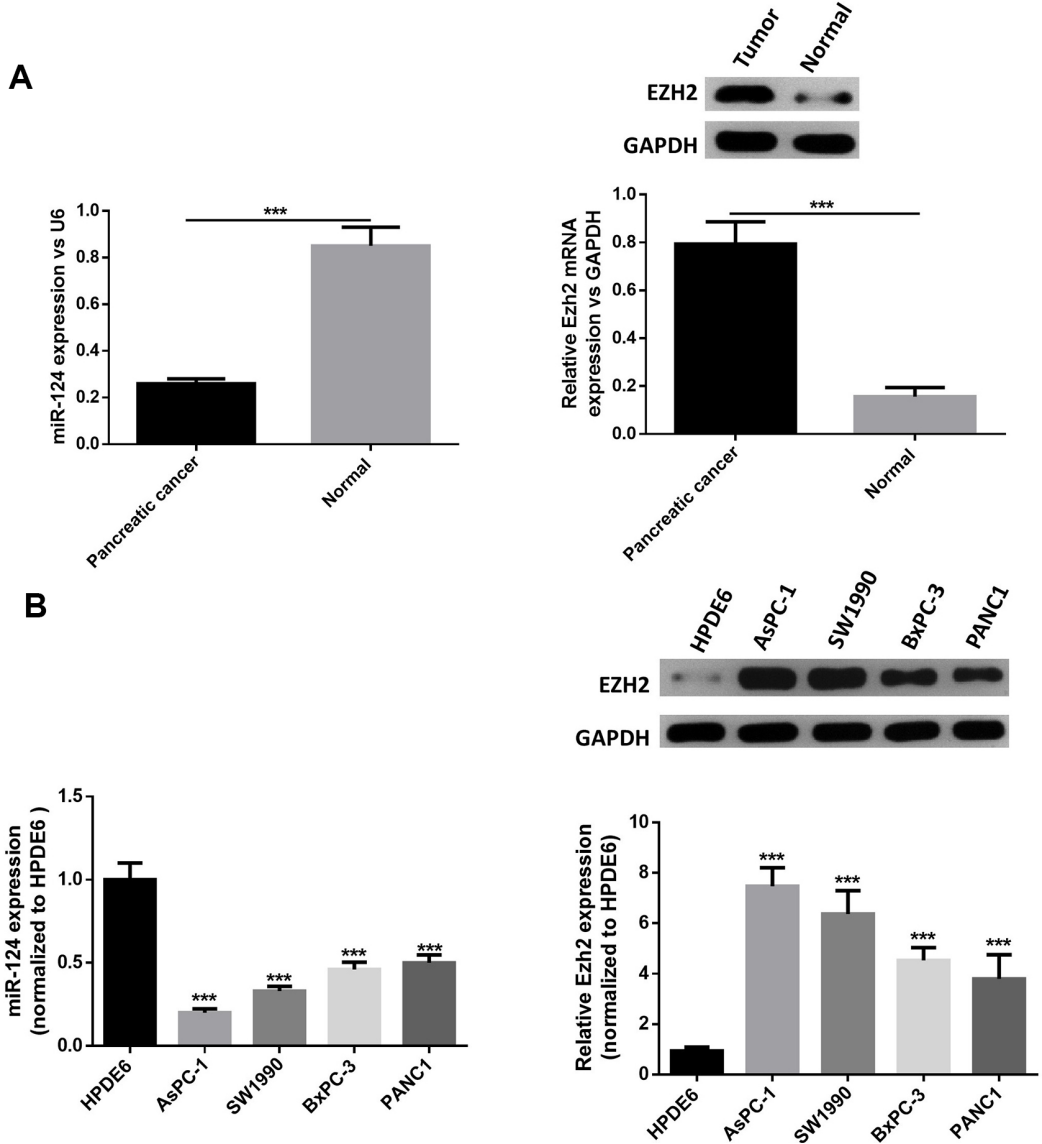
**MiR-124 expression was downregulated and the expression of EZH2 was stimulated in pancreatic cancer tissues and cell lines.** Relative expression levels of miRNA-124 or EZH2 in pancreatic cancer tissues (**A**) or pancreatic cancer cell lines (**B**) AsPC-1, PANC1, BxPC-3 and sw1990, and pancreatic duct epithelial cell line HPDE6 were determined by qRT-PCR or western blot. The results were presented as the mean ± standard deviation (SD) of three independent experiments. ***P<0.001, compared with normal tissues or HPDE6 cell lines.

First, RT-PCR was performed to examine the miR-124 and EZH2expression in pancreatic cancer tumor tissues. The results revealed that the miR-124 expression significantly decreased in pancreatic tumors, while the EZH2 expression in pancreatic cancer tissues was remarkably elevated (Figure 1A). Then, the expression levels of miR-124 in pancreatic cancer cell lines AsPC-1, PANC1, BxPC-3 and SW1990, and normal human pancreatic duct epithelial cell line HPDE6were detected, and the results were consistent with those of the clinical samples. Furthermore, the expression levels of miR-124 in each pancreatic cancer cell line were all notably lower, while the EZH2 expression were upregulated than that in HPDE6 (Figure 1B). As the AsPC-1 and PANC1 cell lines in which the expression of miR-124 was the highest or lowest in pancreatic cancer cell lines were used for further researches.

### The overexpression of miR-124 suppressed cell viability and induced cell apoptosis in pancreatic cancer through regulation of EZH2

**Figure 2:**
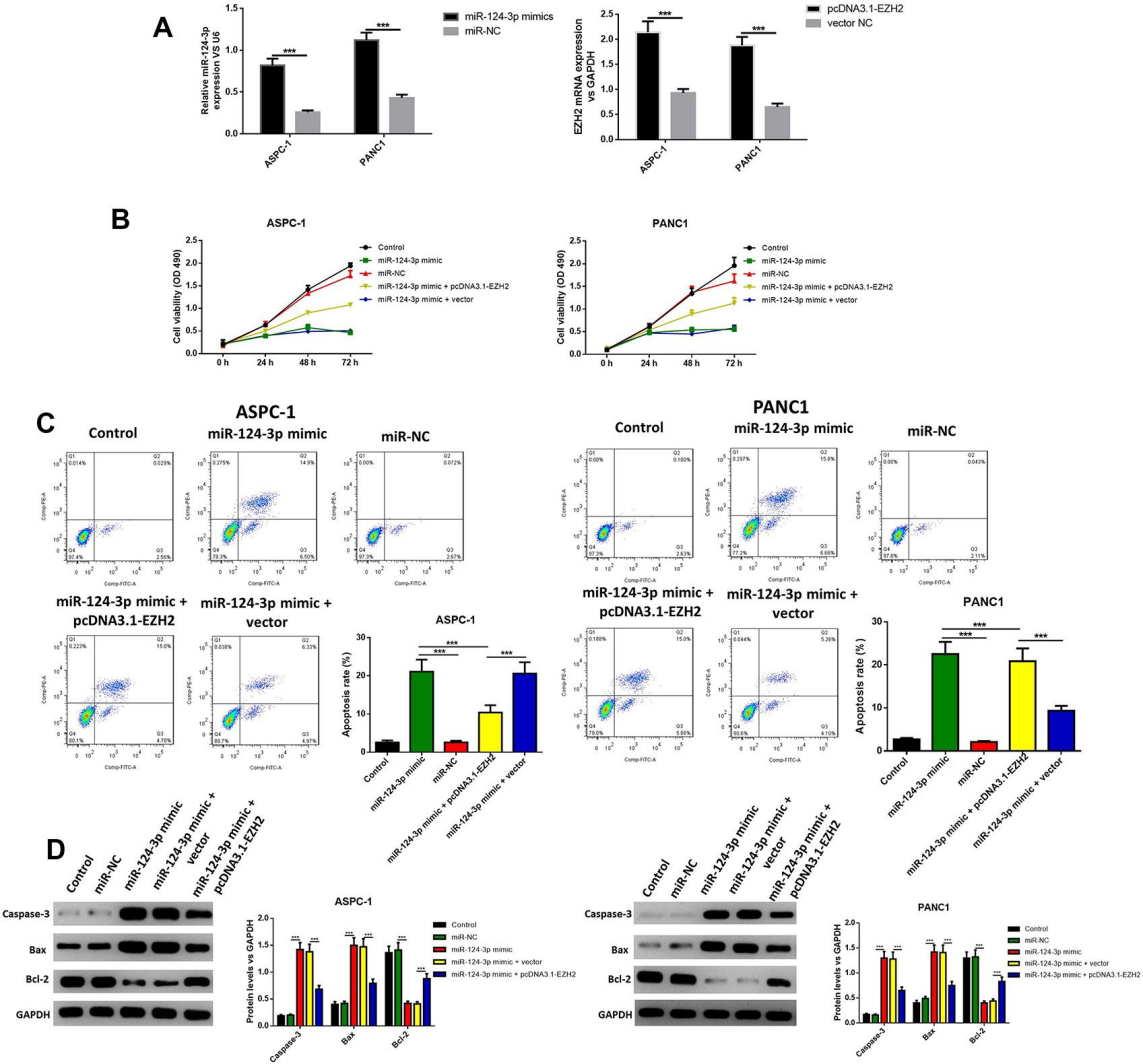
**The miR-124-mimic transfection significantly decreased the cell viability in AsPC-1 and PANC1 cell lines, and induced apoptosis, while these effects were removed by the miR-124 inhibitor or EZH2 overexpression.** Transfection efficacy was determined by RT-qPCR (**A**). Cell viability was determined by MTT (**B**), the apoptosis ratio was determined using Annexin V-propidium iodide (PI) by flow cytometry (**C**), and the expression of cleaved caspase-3, BAX and Bcl2 were determined by western blot (**D**). The results were presented as the mean ± standard deviation (SD) of three independent experiments.

In order to further explore the influence of miR-124 against pancreatic cancer, the miR-124-3p mimic and pcDNA3.1-EZH2 were transferred into AsPC-1 and PANC1 cell. The miR-124 mimic transfection significantly increased the intracellular miR-124 levels, while the transfection of pcDNA3.1-EZH2 remarkably enhanced the expression of EZH2 (Figure 2A). Furthermore, cell viability was examined using the MTT test, and the results indicated that the overexpression of miR-124significantly decreased the cell viability, while the overexpression of EZH2 reversed this effect (Figure 2B). Next, the apoptotic ratio of AsPC-1 and PANC1 after the miR-124 mimic or pcDNA3.1-EZH2 transfection was determined by flow cytometry. The results revealed that the overexpression of miR-124 remarkably induced cell apoptosis in both cell lines, while overexpression of EZH2 reversed the effects (Figure 2C). Western blot was used to detect the expression levels of pro-apoptotic caspase-3, BAX and anti-apoptotic Bcl-2, and the results confirmed that the miR-124 mimic tempted the apoptosis in pancreatic tumor cells, which was reversed by overexpression of EZH2 (Figure 2D). These results suggested interaction between miR-124-3p and EZH2 might regulate cell viability and apoptosis of pancreatic cancer cells.

### Overexpression of miR-124 reduced the invasion, migration and epithelial mesenchymal transition (EMT) of pancreatic cancer cells through regulation of EZH2

**Figure 3:**
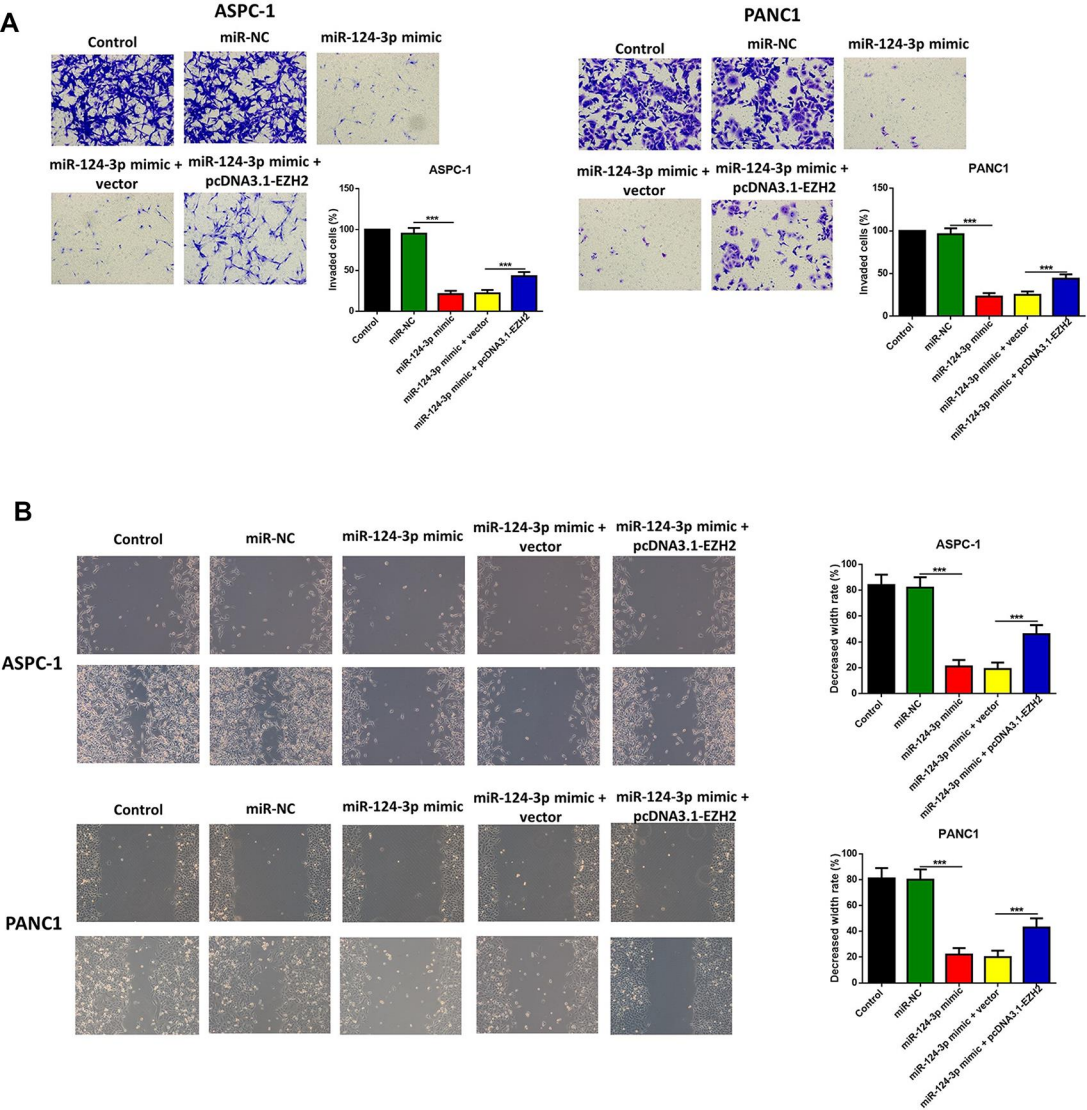
**The miR-124-mimic transfection inhibited the invasion, migration of AsPC-1 and PANC1 cells, which were partly reversed by the miR-124 inhibitor or EZH2 overexpression.** The capability of cell invasion was determined by transwell assay (**A**). The wound-healing assay was used for measurement for cell migration (**B**).

**Figure 4:**
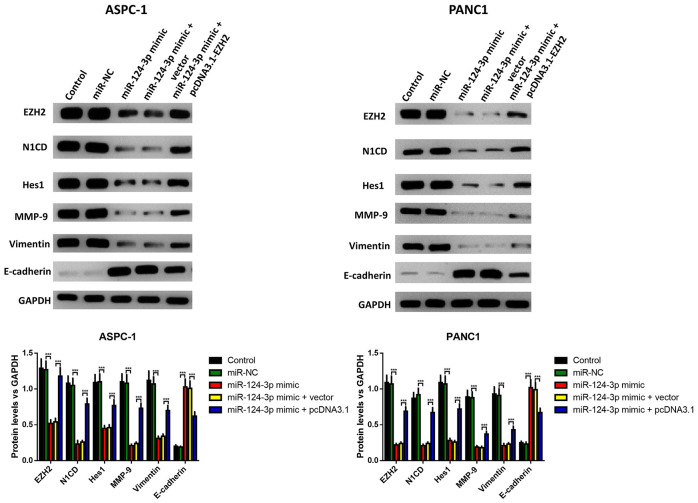
**The miR-124-mimic transfection inhibited the EMT of AsPC-1 and PANC1 cells, which were partly reversed by the miR-124 inhibitor or EZH2 overexpression.** The expression of N1CD, Hes1, MMP-9, E-cadherin and vimentin were determined by western blot. These results were presented as the mean ± standard deviation (SD) of three independent experiments. **P<0.01, compared with the control.

The influence of the miR-124 mimic against the invasion and migration of pancreatic cell lines AsPC-1 and PANC1 were analyzed, and the influence of miR-124 on pancreatic cancer metastasis was investigated. the Transwell assay indicated that cell invasion was remarkably inhibited by overexpression of miR-124-3p, and the wound-healing assay illustrated that the miR-124 mimic-transfected AsPC-1 and PANC1 cells had an obviously lower capability of migration than the control, and all the both effects on cell invasion and migration were reversed by co-transfection with pcDNA3.1-EZH2 (Figure 3A, 3B). Furthermore, the expression levels of N1CD, Hes1, MMP-9 and Vimentin were all downregulated, while E-cadherin had an increased expression level in the miR-124 mimic transfected cell lines, which was reversed by overexpression of EZH2 (Figure 4).

### MiR-124 directly inhibited the expression of EZH2 in pancreatic cancer cells

**Figure 5:**
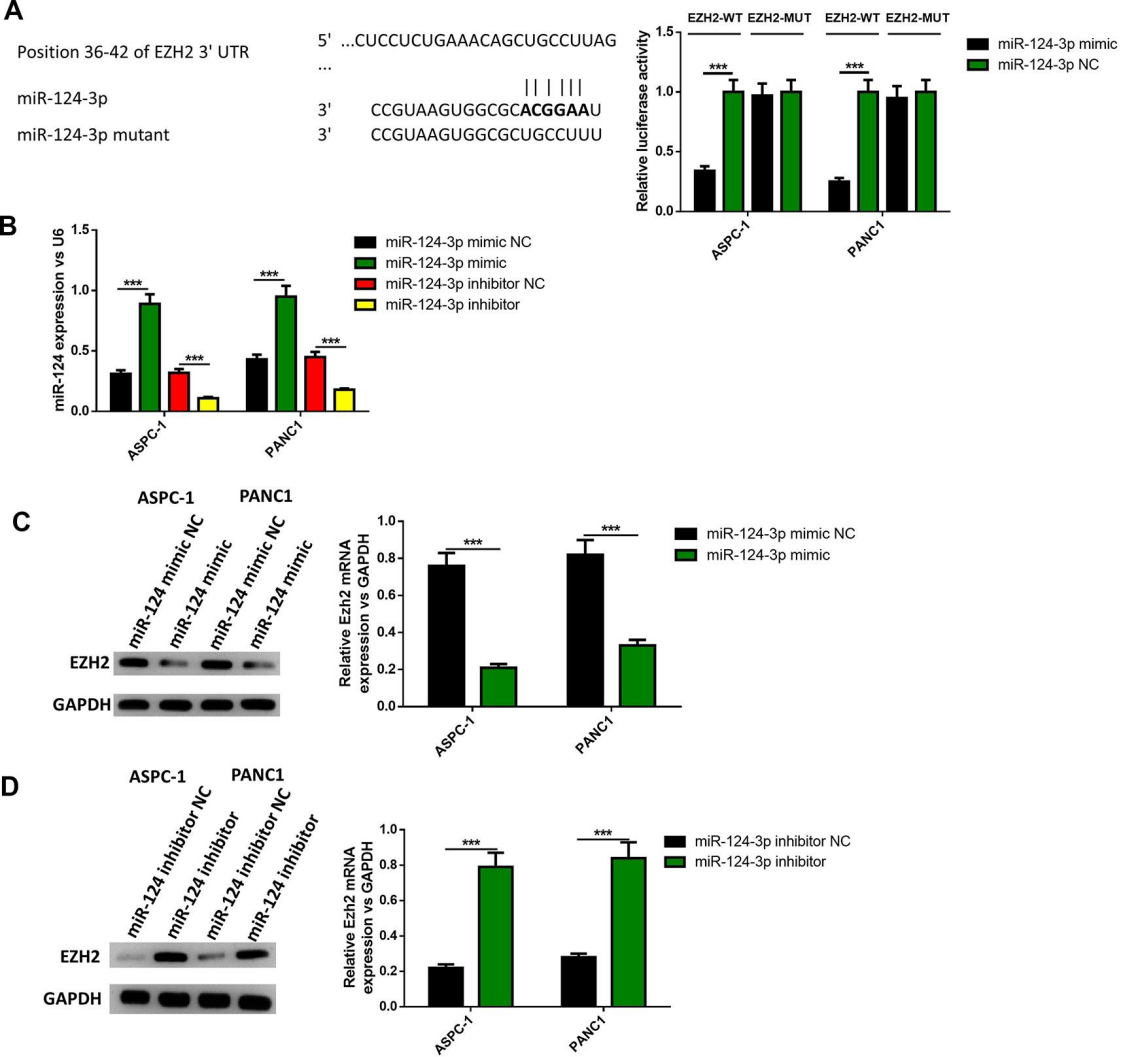
**EZH2 is a target of miR-124.** The binding site between miR-124 and EZH2 was predicted (**A**), dual-luciferase reporter systems were built (**B**) and the report data illustrated that EZH2 is the target gene of miR-124-3p. The expression of EZH2 in miR-124 inhibitor-treated cells were determined by western blot (**C**, **D**). These results were presented as the mean ± standard deviation (SD) of three independent experiments. **P<0.01.

Since the overexpression of EZH2 can partly reverse the influence of the miR-124 mimic on the apoptosis, invasion and migration of pancreatic cells, it was determined whether EZH2 is a direct miR-124 target using luciferase reporter systems. As shown in Figure 5A, the predicted binding site between miR-124 and EZH2 was obtained from targetscan software. The relative activity of luciferase obviously declined in miR-124 mimic-transfected cells and was significantly increased in miR-124 inhibitor-transfected cells in WT-EZH2, while no significant difference was found in MUT-EZH2 in both AsPC-1 and PANC1 cell lines (Figure 5B). Further researches showed miR-124 mimic transfection obviously declined the mRNA and protein levels of EZH2 in the AsPC-1 and PANC1 cell lines, while inhibition of miR-124 led to opposite results (Figure 5C, 5D). These findings indicate that miR-124 has the capability to directly inhibit the expression of EZH2.

### Preparation of BM-MSC-derived exosomes for miR-124 delivery

**Figure 6:**
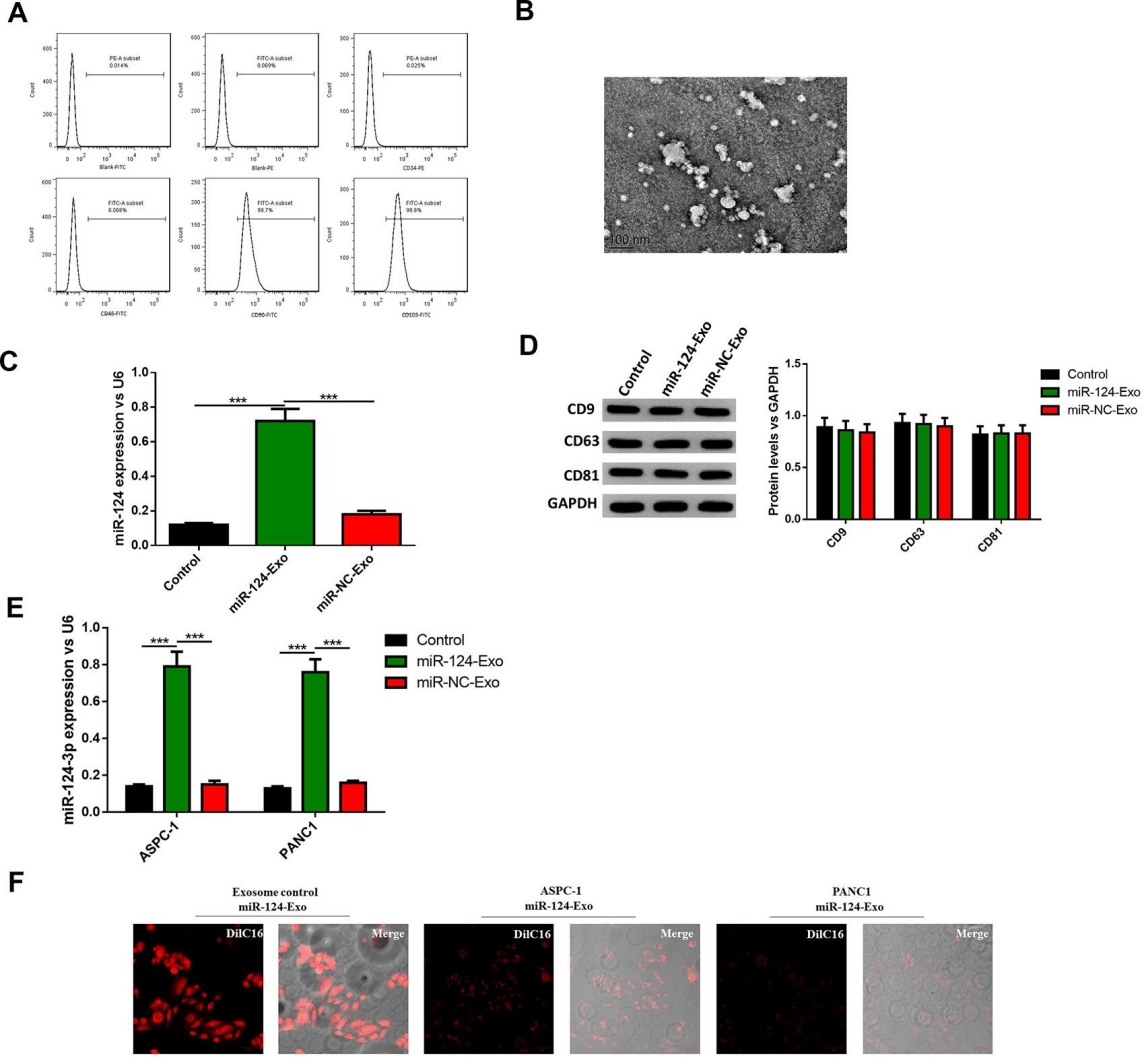
**Exosomes were extracted from BM-MSCs and delivered miR-124 into pancreatic cancers.** The BM-MSCs were identified using flow cytometry (**A**) since CD90 and CD105 were positive, and CD34 and CD45 were negative. The extracted exosomes were examined by transmission electron microscopy (**B**) The expression level of miR-124 in BM-MSCs were determined by RT-PCR (**C**) The expression level of miR-124 in exosomes extracted from the BM-MSCs were determined by RT-PCR, and the protein level of CD9, CD63 and CD81 were determined by western blot (**D**) The expression levels of miR-124 in miR-124-exo co-cultured AsPC-1 or PANC1 cells were determined by RT-PCR (**E**) and cells were stained using Dil C16 dye (**F**). These results present the mean ± standard deviation (SD) of three independent experiments. ***P<0.001.

To further investigate whether BM-MSC-derived exosomes can be vehicles to deliver miR-124 into pancreatic cancer cells, BM-MSCs were extracted from the bone marrow of mice, and sub-cultured in conditional medium. The results illustrated that BM-MSCs possessed different markers, including positive mesenchymal markers CD90 and CD105, and endothelial markers CD34 and CD45 (Figure 6A). Then the morphology of exosomes was observed (Figure 6B) and the diameter of these vesicles was approximately 30-100 nm, which is a typical range for exosomes. It was hypothesized that BM-MSCs-derived exosomes have the capability to secrete miR-124 *via* exosomes thus the exosomes were transfected with the miR-124 mimic (miR-124-Exo) or miR-NC (miR-NC-Exo). Results showed the expression level of miR-124 was remarkably higher in miR-124-Exo than miR-NC-Exo (Figure 6C). In order to further identify these BM-MSC-derived exosomes, the expression levels of CD9, CD63 and CD81 were measured by western blot. The results illustrate that these markers were all expressed in exosomes isolated from natural exosomes, miR-124Exo and miR-NC-Exo (Figure 6D), demonstrating that these exosomes were successfully isolated from the medium. Next, the above exosomes were co-cultured with either AsPC-1 or PANC1 cells and the expression level of miR-124 in miR-124-exo was obviously higher than that in miR-NC-exo or control-exo (Figure 6E). Further DilC16 staining also demonstrated the cell membrane of AsPC-1 or PANC1 cells showed red stain in exosomes co-cultured cells (Figure 6F), indicating that exosomes could bind to receptor cells. All these results suggested that BM-MSC-derived exosomes might deliver miR-124 into pancreatic cancer cells.

### The miR-124-carried BM-MSC-derived exosomes suppressed the proliferation, invasion, migration and EMT of pancreatic tumor cells, and induced apoptosis in pancreatic cancer cells

**Figure 7:**
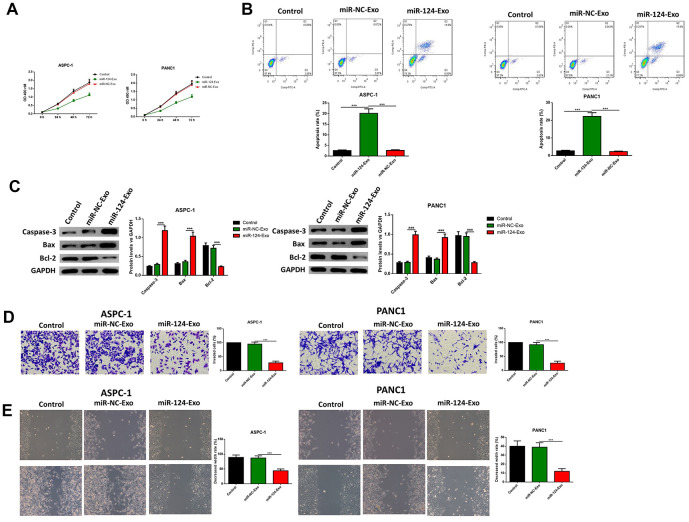
**Exosomes-delivered miR-124 inhibited the proliferation, invasion, migration and induced the apoptosis in pancreatic cancers.** The effects of BM-MSC-derived exosomes on AsPC-1 or PANC1 proliferation, apoptosis was determined using MTT (**A**) Annexin V-propidium iodide (PI) by flow cytometry (**B**) respectively and western blot was used to measure the apoptosis related proteins (**C**) Transwell invasion assay was conducted (**D**) and the wound-healing assay was used for migration assay (**E**). These results present the mean ± standard deviation (SD) of three independent experiments. ***P<0.001.

**Figure 8:**
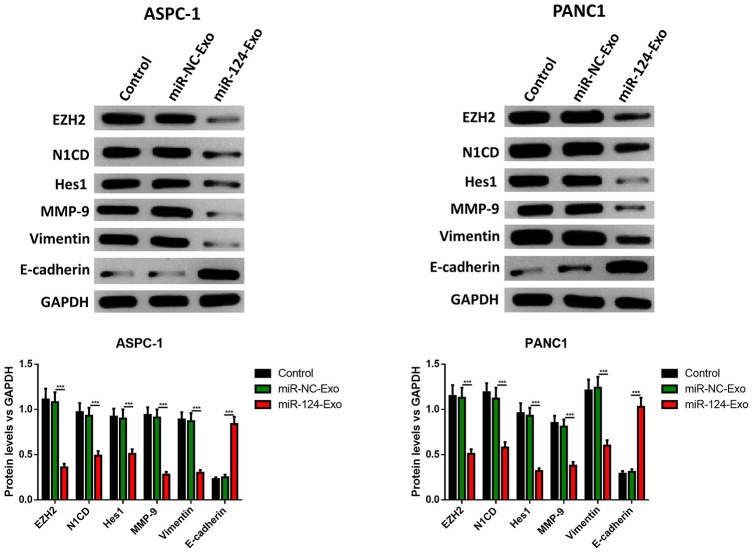
**Exosomes-delivered miR-124 inhibited the EMT in pancreatic cancers.** The protein levels of EZH2, N1CD, Hes1, MMP-9, vimentin and E-cadherin were determined by western blot (D). These results present the mean ± standard deviation (SD) of three independent experiments. ***P<0.001.

AsPC-1 or PANC1 cells were co-cultured with miR-124-exo, and the cell viability, apoptosis, invasion, migration and EMT related proteins were measured. These results indicated that miR-124-carried exosomes slowed down the proliferation of pancreatic cancer cells (Figure 7A). The flow cytometry analysis results showed that the presence of miR-124-exo increased the ratio of apoptotic cells, which was confirmed by the alteration of caspase-3, Bax and Bcl-2 (Figure 7B, 7C). The wound-healing assay, and Transwell assay revealed that miR-124-exo declined the capability of invasion and migration for pancreatic cancer cells (Figure 7D, 7E). Western blot was used to determine the expression levels of EMT related proteins EZH2, N1CD, Hes1, MMP-9, vimentin and E-cadherin. It was found that the miR-124-exo co-culture upregulated the expression of E-cadherin, and the expression of EZH2, N1CD, Hes1, MMP-9 and Vimentin were all reduced in both cell lines (Figure 8). These results implied that miR-126124-carried BM-MSC-derived exosomes suppressed the proliferation, invasion, migration and EMT of pancreatic tumor cells, and induced apoptosis in pancreatic cancer cells.

### The miR-124-carried BM-MSC-derived exosomes sensitized pancreatic cancer cells to chemotherapy *in vitro* and *in vivo*

**Figure 9:**
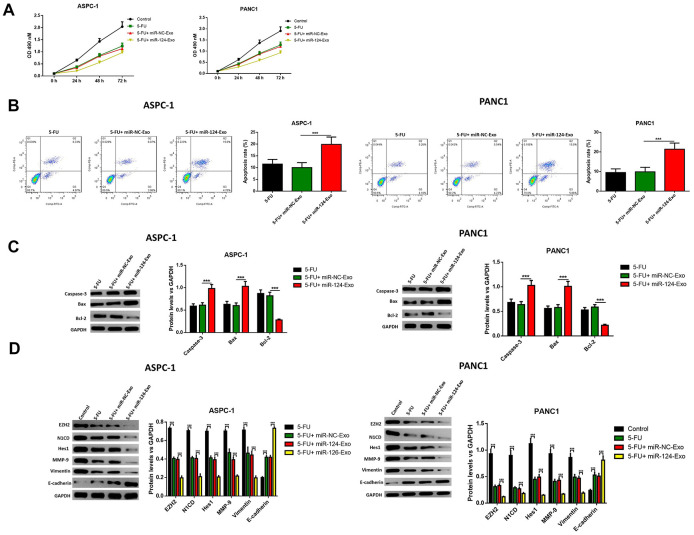
**Exosomes-delivered miR-124 enhanced the chemotherapy on pancreatic cancers.** The effects of BM-MSC-derived exosomes on AsPC-1 or PANC1 cell viability (**A**) were measured by MTT and apoptosis were determined using AnnexinV-propidium iodide (PI) by flow cytometry (**B**), while the apoptosis related protein (**C**) levels of caspase-3, Bax, Bcl-2 and EZH2 and EMT related proteins (**D**) N1CD, Hes1, MMP-9, vimentin and E-cadherin were determined by western blot. These results present the mean ± standard deviation (SD) of three independent experiments. ***P<0.001.

Then, *in vitro* study was conducted to determine whether exosomal miR-124 can increase the sensitivity of pancreatic cancer cells to 5-FU. For AsPC-1 or PANC1 cells, which were treated with 5-FU or 5-FU plus miR-124-exo, flow cytometry and western blot were applied to detect the cell status. The results revealed that 5-FU treatment alone has the capability to tempt apoptosis, and suppress cell viability in pancreatic tumor cells, and that miR-124-exo co-culturing with 5-FU treatment remarkably enhanced the effects of 5-FU on cell apoptosis and viability (Figure 9A–9C). Besides, miR-124-exo also enhanced the effects of 5-FU on EMT related proteins and EZH2, in which miR-124-exo remarkably enhanced the expression of E-cadherin, and decreased the expression of EZH2, N1CD, Hes1, MMP-9 and Vimentin (Figure 9D).

**Figure 10:**
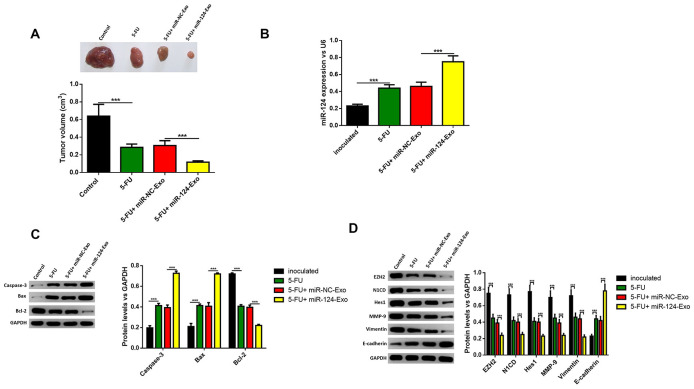
**Exosomes-delivered miR-124 enhanced the chemotherapy on pancreatic tumors *in vivo*.** MiR-124-carried exosomes enhanced the anti-tumor effects of 5-FU on pancreatic cancer (**A**) The expression of miR-124-3p was remarkably higher in miR-124-EXO group (**B**) And the apoptosis related protein (**C**) levels of caspase-3, Bax, Bcl-2 and EZH2 and EMT related proteins (**D**) N1CD, Hes1, MMP-9, vimentin and E-cadherin on pancreatic tumors were determined by western blot. These results present the mean ± standard deviation (SD) of three independent experiments. ***P<0.001.

The above results were confirmed *in vivo*. MiR-124-exo were injected into mice treated with 5-FU, and the tumor size was narrowed, when compared with mice treated with 5-FU alone (Figure 10A). The expression of miR-124-3p was also remarkably higher in miR-124-exo group (Figure 10B). Then, the proteins were extracted from the tumor tissues, and the expression levels of caspase-3, BAX and E-cadherin increased, while the expression of Bcl-2, EZH2, N1CD, Hes1, MMP-9 and vimentin were inhibited in tumors from miR-124-exo and 5-FU co-treated mice, when compared to 5-FU treatment alone (Figure 10C, 10D), which was consistent with that in the *in vitro* experiments.

## DISCUSSION

In the latest decades, the function of miRNAs has attracted increasing attention, since the development of predictive tools suggest that the expression of thousands of genes are regulated by miRNAs [20]. MiRNAs were found to play physiologic and pathologic roles in humans, and affect the metabolism, immune response and other functions in metabolic diseases and cancer [21–23]. In pancreatic cancer, more than 20 miRNAs were found to be associated with proliferation, metastasis, apoptosis, or chemosensitivity [9, 23]. Among these, miR-124 attracted increasing attention. In the present study, it was found that the miR-124 expression in pancreatic cancer or pancreatic cancer cell lines all decreased, and overexpression of miR-124 delivered by BM-MSC derived exosomes suppressed cell viability, invasion and migration, as well as induced cell apoptosis in pancreatic cancer through regulating EZH2.

MiR-124 is considered as a tumor suppressor in many cancers. It was found miR-124 inhibited cell viability and invasion of HR-HPV-positive cervical cancer cells by targeting RBG2, which was regulated by lncRNA MALAT1 [24]. Another study also found miR-124 inhibited cancer development in breast cancer [25]. In a recent study, miR-124 was also found to suppress growth, invasion and migration in bladder cancer [26]. Indeed, the present study revealed that the transfection of miR-124 or co-culture with miR-124 that carried exosomes inhibited the cell viability, invasion and migration, as well as EMT in AsPC-1 and PANC1 cells. And the effects might be through regulation of EZH2.

EZH2 has been considered as another target of miR-124 [27]. EZH2 has an important function as a histone methyl transferase to catalyze the methylation of lysine27 of histone H3, and dynamically modifies the structure of chromatin and blocks the gene transcription [28]. Since DNA and histone hypermethylation has been linked to carcinogenesis, and the overexpression of EZH2 was found in numerous cancer forms, including breast cancer, prostate cancer, leukemia and pancreatic tumors, this has been considered to be a potential cancer therapy target [29–32]. A previous study revealed that EZH2 is the target of miR-124 in gastric tumor cells [16]. In the present study, it was found that miR-124 could induce the apoptosis and suppress the cell invasion, migration and EMT of pancreatic cancer cells, and miR-124 and these effects could be partly restored by EZH2 overexpression. These findings suggest that miR-124 presents the anti-cancer role in an EZH2-dependent manner in pancreatic cancer.

Exosomes are defined as 30-100 nm vesicles secreted from multi-vesicular bodies, and are distinguished from other extracellular vesicles [33]. Exosomes have the capability to bear and deliver proteins, phospholipids, cholesterol, mRNA, miRNA and other non-coding RNAs, and play important roles in intracellular communication [19]. In cancer, exosomes may take part in proliferation, metastasis and chemoresistance [17]. At present, the miRNAs in exosomes have attracted increasing attention in cancer diagnosis and therapy. MiRNAs are expressed and sorted into exosomes, and regulate the functions of recipient cells [34]. The miRNAs of exosomes can be employed as circulating bio-markers for numerous cancers, including colorectal tumor, breast tumor, prostate tumor and pancreatic tumor [35–37]. In pancreatic cancer, miR-21, miR-17-5p, miR-10b, miR-23b-3p, miR-196a and miR-1246were identified as potential exosomal miRNA markers for diagnosis and/or prognosis [38–40]. Some exosomal miRNAs were identified as potential cancer therapeutic targets, since these miRNAs may take part in carcinogenesis, metastasis and drug resistance [41, 42]. For instance, miR-301a was found to be correlated to macrophage polarization, and was considered to be a potential treatment target for pancreatic tumors [43]. Recently, exosomal miRNAs with anti-cancer effects were considered as novel cancer therapy candidates [44]. In the present study, it was demonstrated that BM-MSC-derived exosomes have the capability to deliver miR-124 into pancreatic tumor cells, and that miR-124-carried exosomes suppressed the proliferation, metastasis and EMT, and enhanced the chemosensitivity to 5-FU *in vitro* and *in vivo*.

The present study also has some limitations. The number of tissue samples is small and the clinical significance of miR-124 in pancreatic cancer is unclear. Besides, deeper insights are still needed to reveal the underlying molecular mechanisms for miR-124 in pancreatic cancer. All these need more *in vitro* and *in vivo* studies to confirm.

## CONCLUSIONS

MiR-124-carried BM-MSC-derived exosomes are novel candidates for pancreatic cancer treatment. Determining how to deliver miRNAs with anti-cancer effects to the pancreatic cancer microenvironment remains as a challenge for the application of miRNA-based therapy. These present findings suggest that exosomes derived from BM-MSCs can be considered as a potential miR-124 vehicle in pancreatic cancer treatment.

## MATERIALS AND METHODS

### Tissue samples

Eight pairs of formalin-fixed and paraffin-embedded pancreatic adenocarcinoma tissues and the corresponding non-tumorous pancreatic tissues were collected from Tongji Hospital (Shanghai, China) from the tissue bank of the hospital. All tissue samples in the present were collected before chemotherapy immediately after resection. The present study was approved by the Ethics Committee of Tongji Hospital, Medical School of Tongji University (Shanghai, China). Each sample was stained with hematoxylin and eosin (H&E) for histopathological identification.

### Separation of rat mesenchymal stem cells

The bone marrow cells were separated from the whirl bone of male Sprague-Dawley (SD) rats. Then, these marrow cells were cultured in culture medium (MesenPRO RS™ Medium, Gibco; 12746–012) and separated using a BM-MSC Isolation Kit (TBD). Afterwards, these cells were collected, placed in a culture bottle, and culture for 2-3 days without disturbing. Then, the culture medium was changed twice, and the fused monolayer cells were observed at the third generation. The BM-MSCs were identified by flow cytometry.

### Extraction and identification of exomes

For extraction of BMSCs-derived exosomes, BM-MSCs were cultured in culture medium with 10% exosome-depleted FBS before the extraction of exosomes. Then, the culture medium was collected and prepared for exosome collection within 24-48 hours. Next, a BM-MSC solution of 50 mL was placed into a centrifuge tube on ice. Then, the mixture was centrifuged at 4°C with 800×g for 10 minutes to remove the sediment cells. Afterwards, the supernatant was subsequently centrifuged using 100 kDa Millipore ultrafiltration at 12,000×g for 20 minutes at 4°C to remove the cellular debris. The exosomes were separated from the supernatant according to the instructions of the exosome extraction kit.

For identification, the supernatant was collected, centrifuged and resuspended in 200 μl PBS, fixed by with 2% paraformaldehyde and loaded on parafilm. The exosome samples were observed under a transmission electron microscopy (JEM-1400PLUS, Japan) at 100 KV and the NTA software was used to evaluate the size of exosomes. Annexin V-propidium iodide (PI) staining was performed for analysis of CD45-FITC, CD34-PE, CD90-FITC and CD105-PE for the identification of exosomes, according to manufacturer’s instructions, which were analyzed using a BD LSRFortessa with flow cytometry.

### Cell culture and transfection

The human pancreatic cancer cell lines AsPC-1, PANC1, BxPC-3 and SW1990, and normal pancreatic epithelial HPDE6 cells were provided by the Cell Resource Center of Shanghai Institute of Biochemistry. All cells were cultured in RPMI-1640 (Thermo Fisher Scientific, Waltham, MA, USA), which contained 10% fetal bovine serum.

For cell transfection, the miRNA-124-3p mimics and negative control oligonucleotides (10 nM for each) (synthesized by GenePharma Co., Ltd., Shanghai, China) were transfected into the cells using Lipofectamine 2000 reagents (Invitrogen Life Technologies), according to the protocol of the manufacturer. After transfection for 48 hours, cells were collected to conduct the analyses, and determined by qRT-PCR and western blot. For co-incubation of pancreatic cancer cell lines and exomes, the extracted exomes (extracted from 200 mL culture medium) which were transfected with miR-124-3p mimics (miR-124-exo), or mimics NC (miR-NC-exo) (10 nM for each) were co-incubated with AsPC-1 and PANC1 cell lines.

### MTT Assay

For MTT Assay, cells (1×10^4^ mL^-1^) were seeded on each well of a 96-well plate at 37°C in an incubator containing 5% CO_2_ overnight. After incubation for 24 hours at 37°C, 20 μL of MTT was added to each well, and incubation was continued for another four hours. Then, the medium was removed and 100 μL of dimethyl sulphoxide (DMSO) was added. Afterwards, the optical density (OD) of each well at 490 nm was recorded using an enzyme-linked immunodetector (Spark 10M, TECAN).

### RNA separation and Real-time reverse transcription-polymerase chain reaction (RT-PCR)

The total RNA extracted from cultured cells were detected using TRIzol reagent (Invitrogen, Carlsbad, CA, USA). Then, the total RNA of the total tissues extracted from the FFPE tissue slice were detected using an miRNeasy FFPE kit (Qiagen, Valencia, CA, USA), according to manufacturer's instructions. The integrity of the RNA was detected by electrophoresis with 1.5% agarose gel, and the concentration of the RNA was detected using NanoDrop 1000 (Thermo Fisher Scientific, Waltham, MA, USA). The synthesis of the cDNA was conducted according to the recommendation of the manufacturer. Briefly, the RNA was reverse-transcribed (RT) using a ReverAid First Strand cDNA kit, in combination with a stem-loop primer for miRNA-124. Then, U6 snRNA or GAPDH was used as the reference genes. Then, the cDNA products were diluted at 20×, and the 10-μL PCR mixture contained 1 μL of diluted RT product, 5 μL of SYBR-Green Master Mix, 2 μL of RNase-free water, 1 μL of forward primer, and 1 μL of reverse primer. These amplifications were performed in a 96-well plate at 95°C for 10 minutes, followed by 40 cycles of 95°C for 15 seconds and 60°C for one minute. Each sample was run in triplicate. The relative miRNA-124 and mRNA expression were expressed using the 2^-ΔΔCt^ method.

### Western blot assay

Briefly, the collected protein samples were extracted using RIPA buffer, and one-fourth of each protein sample of the concentrated sodium dodecyl sulfate-polyacrylamide gel electrophoresis (SDS-PAGE) protein buffer solution was added, and boiled for five minutes to make the protein denature. Then, the protein samples were separated by SDS-PAGE gel and transferred to nitrocellulose membranes at 200 mA for 90 minutes. After blocking the membranes with 5% low-fat milk powder, which contained Tris-buffered saline-Tween 20 solution, at room temperature for two hours, the membranes were incubated with primary antibodies at 4°C overnight. All primary antibodies (CD9, CD63, CD81, Ezh2, N1CD, Hes1, MMP-9, E-cadherin, vimentin caspase-3, Bax and Bcl-2, Abcam) were diluted at 1:500. After removing the primary antibody, the membranes were incubated with the appropriate horseradish peroxidase-labeled secondary antibody (diluted at 1:5,000, Abcam) for one hour at room temperature. Then, the bound antibodies were viewed using enhanced chemiluminescence reagents. The protein bands were obtained using a multi-function imager, and semi-quantified using the ImageJ software.

### Wound healing assay

124Cells were cultured in a 24-well plate up to confluence. Then, these were wounded by dragging a 200-μl pipette tip through the monolayer. Afterwards, all cells were washed to remove the cellular debris, which allowed cells to migrate for 24 hours. Images were recorded at 0 and 24 hours after wounding under an Eclipse Ti-U (Nikon, Kanagawa, Japan) inverted microscope. The relative surface traveled by the leading edge was evaluated using the Image-Pro Plus 6.0 software, and each independent experiment was conducted for three times.

### Transwell assays

The cell invasion assay was performed using Matrigel-coated Transwell chambers with a pore size of 8 μm. At 48 hours after transfection, these cells were seeded onto the upper chambers at a final concentration of 5×10^4^ cells/well, and cultured in 100 μL of serum-free medium. The lower chambers contained 700 μL of medium with 10% FBS. After 40 hours of incubation, these cells were allowed to migrate or invade through the filter into the lower side of the chamber, and were fixed and stained with crystal violet for 30 minutes. Then, the number of cells was counted under a microscope, and counted using Image-Pro Plus version 6.0. The invaded cell rates were calculated as invaded cells in test group/blank control group.

### Luciferase reporter assay

For luciferase detection, a fragment of the EZH2 gene 3'UTR that contains the miR-124 binding site was amplified in the pMIR-REPORT vector (Ambion, Austin, TX, USA), and cloned to the downstream of the firefly luciferase gene. The mutation vector was generated by cloning the mutated fragment into the pMIR-REPORT vector. Then, AsPC-1 and PANC1 cells were cultured into a 96-well plate to transfect with the miR-NC and miR-124 mimics. After incubation for 48 hours, these cells were lysed, and luciferase activity assay was performed using the Dual Luciferase Reporter Assay System (Promega, Madison, WI, USA).

### Tumor xenograft assay in BALB/c Nu Nu mice

For tumor xenograft assay, 24 male BALB/C nude mice (4~6 weeks, 12±2 g) were purchased from SJA Laboratory Animal company (Hunan, China). All animals were kept in a light-controlled room under a 12 h/12 h light/dark cycle and controlled temperature (23-25°C), and had free access to food and water. In particular, any effort was put to avoid unnecessary pain of the animals. The whole study was approved by the Institutional Animal Care Committee at Tongji Hospital, Medical School of Tongji University (Shanghai, China). Briefly, the AsPC-1 cells, 1×10^5^) which were co-incubated with miR-124-exo or miR-NC-exo were inoculated in mice at the subcutaneous right part. Afterwards, mice were sacrificed after inoculating for 35 days, and analyzed by tumor weight. Each group included 6 mice. For treatment of 5-FU, each mouse received 5 mg 5-FU around the tumor bearing site.

### Statistical analysis

All experimental data were independently repeated for at least three times. One-way analysis of variance (ANOVA) followed by Tukey post hoc test. was used to analyze the data and comparison between two groups was performed using the Student t-test., which was expressed as mean ± standard deviation (SD). Statistical significance was defined as *P*<0.05.

### Ethics approval and consent to participate

The present study was approved by the Ethics Committee of Tongji Hospital, Medical School of Tongji University (Shanghai, China), and all patients provided a signed informed consent.

### Availability of data

All data generated or analyzed during this study were included in the published article.
